# The effectiveness of a preventive health program and vitamin D status in improving health-related quality of life of older Canadians

**DOI:** 10.1007/s11136-015-1103-7

**Published:** 2015-08-18

**Authors:** J. P. Ekwaru, A. Ohinmaa, Paul J. Veugelers

**Affiliations:** School of Public Health, University of Alberta, 3-50 University Terrace, 8303 – 112 Street, Edmonton, AB T6G 2T4 Canada

**Keywords:** Health-related quality of life, Vitamin D, Public health, Disease prevention, Intervention, Nutrition

## Abstract

**Purpose:**

To assess the effectiveness of a preventive health program and vitamin D status in improving the health-related quality of life (HRQOL) of older residents of Canada.

**Design:**

We analyzed baseline and follow-up data of 2119 volunteers of a community program that promotes healthy lifestyles and encourages vitamin D supplementation. We examined the program effect on each of the five dimensions of the EQ-5D-5L, HRQOL score, and quality-adjusted life years (QALYs) using multivariable regression methods. We further examined the specific contribution of vitamin D status as quantified by serum 25-hydroxyvitamin D (25(OH)D).

**Results:**

Problems with mobility, usual activities, pain/discomfort, and depression/anxiety were reported less during follow-up compared to baseline. On average, participants’ HRQOL had improved by 0.018 units at 6 months and 0.025 units at 1 year of follow-up. Improvements in vitamin D status were independently associated with improvements in HRQOL and in QALYs. As per 25 nmol/L increase in 25(OH)D, there was a 0.002 increase in HRQOL and a 0.001 increase in QALYs.

**Conclusions:**

This study documents the benefits of a real-world preventive health program to HRQOL. It is the first to reveal that improvements in vitamin D status parallel improvements in HRQOL among healthy community dwellers. The study further suggests that the preventive health program and supplementation with vitamin D are cost-effective interventions.

## Introduction

Vitamin D has been shown to benefit bone health and to reduce the burden of various diseases [1]. To mediate the health benefits, the Institute of Medicine and Health Canada recommend a vitamin D intake of 600 IU per day for adults and 800 IU per day for those above the age of 70 years [2,3]. These intakes are assumed to be sufficient to achieve an adequate serum 25-hydroxyvitamin D (25(OH)D) concentration, the established measure of vitamin D status. However, recent studies have suggested that the intake needed to achieve an adequate vitamin D status varies by weight status [4–6].

In Canada, diets contribute an estimated 232 IU of vitamin D per day [7] and, given its Northern latitude, cutaneous synthesis of vitamin D by sun exposure is limited [8]. Despite the Health Canada recommendations, vitamin D deficiency and insufficiency continue to be prevalent [9]. Specifically, the Canadian Health Measures Survey had shown that 4.1 and 10.6 % of Canadians aged 6–79 years had serum 25(OH)D concentrations of less than 27.5 and 37.5 nmol/L, respectively [9].

For older populations, several vitamin D supplementation studies have suggested, though not consistently, less functional limitation [10], prevention of falls [11–13], reductions in fractures [14–16], and benefits to mental health [17, 18]. Based on a meta-analysis, Bischoff-Ferrari et al. [12] had recommended vitamin D supplementation to achieve a reduction in falls. The above studies suggest a relationship between vitamin D and objectively measured health conditions. Health-related quality of life (HRQOL) aims to quantify the subjective experiences of the consequences of these health conditions. Huang et al. attributed improvements in quality of life along with improvements in pain and sleep to vitamin D supplementation in a case series of patients with chronic pain [19]. For a cross-sectional sample of older healthy volunteers, we recently showed that vitamin D status was positively associated with HRQOL [20]. In the latter study, we acknowledged the issue of reverse causality and recommended that intervention studies are needed to show whether vitamin D supplementation leads to improvements in HRQOL [20].

In the present study, we examine the effect of a preventive health program that encourages vitamin D supplementation on HRQOL among older residents of Canada. This seems particularly important to Canadians given the relatively high latitude and consequent reliance on vitamin D from diet and supplements.

## Methods

This is a real-world evaluation of information gathered at baseline and follow-up visits from volunteer participants of a preventive health program by the Pure North S’Energy Foundation (PN). PN, a charitable, not-for-profit organization, provides lifestyle counseling and encourages vitamin D and multivitamin supplementation as described in more detail elsewhere [21–23]. As of August of 2012, PN started the recruitment of older residents of the city of Calgary, Alberta, Canada. PN advertised their program for seniors through local newsletters and through the distribution of flyers in senior homes and community centers. PN organized weekly information meetings after which attendees could elect to sign up to enroll in the program. At their baseline and follow-up visits, participants completed a survey, had their body height and weight measured, and had their blood drawn for the assessment of serum 25-hydroxyvitamin D (25(OH)D), a measure of vitamin D status. Until April 2013, 25(OH)D was measured with the Liaison (chemiluminescent reaction) and, after that with a liquid chromatography, tandem mass spectrometry (LC/MS–MS) method. The correlation for 25(OH)D between methods was 0.801 (*n* = 3015, *p* < 0.001).

The survey included the five-level EQ-5D (EQ-5D-5L) to measure health-related quality of life (HRQOL) [24]. The EQ-5D consists of a five-dimensional descriptive system asking whether participants have (1) no problems; (2) slight problems; (3) moderate problems; (4) severe problems; or (5) extreme conditions or are unable to perform, or extreme conditions are fully constrained or restricted, with each of the following: (1) mobility; (2) self-care; (3) usual activities; (4) pain or discomfort; and (5) anxiety or depression [24]. HRQOL scores are based on responses to each of the five dimensions and were derived from the US value sets [25]. The EQ-5D is an established and validated instrument [24] with the major advantages of being short and easy to complete [26].

The survey further included questions on age, gender, perceived health, and income. Individuals and couples with an annual income of less than $25,000 and of less than $41,000, respectively [27], were considered as low income. Body mass index (BMI) was calculated on the basis of the measured heights and weights (weights in kilograms divided by the square of height in meters). Individuals with a BMI of 25 or more were considered overweight, and those with a BMI of 30 or more were considered obese [28].

Serum 25(OH)D is the established proxy for vitamin D status. Some have suggested that individuals with serum 25(OH)D concentrations below 50 nmol/L should be considered vitamin D deficiency, and those with serum 25(OH)D concentrations of 50 or more and less than 75 nmol/L be considered vitamin D insufficiency [29]. As recent studies have suggested extra-skeletal benefits, such as reduction in colorectal cancer and cardiovascular disease risk, for individuals with serum concentrations of 75 nmol/L or more [1, 30, 31], and where sufficient number of observations with serum concentrations above 75 nmol/L are observed, we further categorized serum concentrations into ≥75 and <100 nmol/L, ≥100 and <125 nmol/L, and ≥125 nmol/L. Where the above categorizations of vitamin D status provide some ease in interpretations, we did confirm our analysis while considering vitamin D status as a linear covariate.

As descriptive statistics we present the changes in the reporting of problems in the five EQ-5D dimensions and in the HRQOL score between baseline and 6 months of follow-up and between baseline and 1 year of follow-up (also referred to as temporal changes). The changes over 6 months of follow-up have the advantage of a larger sample size, and the changes over 1 year of follow-up have the advantage of longer intervention. We used a Chi-square test to test for the significance of temporal changes in the prevalence of problems and a one sample *t* test to test whether the mean temporal changes in HRQOL score were significantly different from zero. We applied univariate and multivariable regression to identify determinants of the temporal changes in HRQOL scores at 6 months and at 1 year. In addition to quantify the temporal changes at 6 months and at 1 year, we fitted an overall linear mixed-effect model for changes in HRQOL over time in the program using all observations regardless of when the observation was made. In all multivariable regression models, we adjusted for age, gender, body weight status, and baseline HRQOL. Furthermore, we examined the importance of vitamin D status for temporal changes in HRQOL scores by considering (1) the baseline serum 25(OH)D concentrations and (2) the difference between the follow-up and baseline serum 25(OH)D concentrations (also referred to as the temporal increase in serum 25(OH)D) as determinants of temporal changes in HRQOL scores.

Quality-adjusted life years (QALY’s) were estimated by the area under the HRQOL curve method [32].

PN anonymized their data prior to forwarding it to the University of Alberta for statistical analysis. The analysis was conducted using STATA version 12 (College Station, Texas). The Human Research Ethics Board of the University of Alberta approved the data access and analysis for this study.

## Results

A total of 2119 participants had both baseline and follow-up assessments of HRQOL and serum 25(OH)D. Baseline characteristics of the 2119 participants are presented in Table 1. Of the 2119 participants, 1795 (84.7 %) and 761 (35.9 %) had follow-up assessments at 6 months (4–8 months) and 1 year (9–15 months), respectively.

**Table 1:** Baseline characteristics of 2119 participants with a baseline and follow-up assessments of HRQOL

Variable	*N*	%	Mean	SD
Gender				
Female	1374	64.8		
Male	745	35.2		
Baseline age				
50–59	409	19.3		
60–69	1011	47.7		
70–79	558	26.3		
80+	141	6.7		
Baseline income				
Other	1098	51.8		
Low income	1021	48.2		
Baseline HRQOL	2119		0.814	0.118
Baseline serum 25(OH)D (nmol/L)	2119		93.449	35.916
Baseline serum 25(OH)D (nmol/L)				
≥50, <75 nmol/L	491	23.2		
<25 nmol/L	11	0.5		
≥25, <50 nmol/L	135	6.4		
≥75, <100 nmol/L	722	34.1		
≥100, <125 nmol/L	447	21.1		
≥125 nmol/L	313	14.8		
Baseline self-reported health				
Very good or excellent	857	40.4		
Good	798	37.7		
Poor or fair	373	17.6		
Baseline weight status				
Underweight or normal weight	715	33.7		
Overweight	796	37.6		
Obesity	561	26.5		

Temporal changes in the prevalence of problems in the five EQ-5D dimensions and the HRQOL score are presented in Table 2. At the follow-up visits, participants reported fewer problems with mobility, usual activities, pain or discomfort, and depression or anxiety compared with what they reported at baseline (Table 2). For the 1795 participants with follow-up assessments at 6 months, the mean HRQOL score was 0.816 at baseline and 0.833 at 6-month follow-up, representing a temporal increase of 0.018 units in 6 months (Table 2). As a result, at 6 months of follow-up, fewer participants would retain in the baseline lower tertile and instead moved to the higher HRQOL tertiles (Table 2). For the 761 participants with follow-up assessments at 1 year, the mean HRQOL score was 0.808 at baseline and 0.832 at 1-year follow-up, representing a temporal increase of 0.025 units in 1 year (Table 2). For participants with follow-up at 1 year, the mean serum 25(OH)D concentration increased from 95 nmol/L at baseline to 129 nmol/L at 1 year of follow-up. At baseline, participants reportedly supplemented on average 2427 IU of vitamin D per day. At one year, this was 7510 IU per day.

**Table 2:** Changes in problems in EQ-5D dimensions, HRQOL score, serum 25(OH)D concentrations from baseline to 6 months and 1 year

Variable	Temporal change from baseline to 6 months (*N* = 1795)				Temporal change from baseline to 1 year (*N* = 761)			
	Baseline	6 months	Change (95 % CI)	*p* value	Baseline	1 year	Change (95 % CI)	*p* value
Problems in EQ-5D dimensions (%)								
Problems with mobility	39.2	35.2	−4.0 (−5.9, −2.0)	0.014	39.4	34.2	−5.3 (−8.5, −2.0)	0.033
Problems with self-care	6.2	6.8	+0.6 (−0.6, 1.7)	0.499	7.1	5.7	−1.4 (−3.3, 0.4)	0.248
Problems with usual activities	34.5	29.0	−5.5 (−11.5, −3.5)	<0.001	35.9	27.6	−8.3 (−11.5, −5.0)	<0.001
Problems with pain and discomfort	76.7	67.9	−8.9 (−11.0, −6.7)	<0.001	81.7	69.0	−12.7 (−16.0, −9.5)	<0.001
Problems with depression and anxiety	47.5	43.1	−4.5 (−6.6, −2.3)	<0.001	51.9	43.8	−8.1 (−11.7, −4.6)	0.001
Any problem	85.1	78.7	−6.5 (−8.3, −4.6)	0.007	88.6	81.5	−7.1 (−9.9, −4.3)	<0.001
Health-related quality of life score, mean	0.816	0.833	0.017 (0.012, 0.021)	<0.001	0.808	0.832	0.025 (0.018, 0.032)	<0.001
Serum 25(OH)D nmol/L, mean	93.0	130.8	37.8 (36.0, 39.6)	<0.001	95.0	129.1	34.2 (31.2, 37.1)	<0.001
Vitamin D supplementation (IU), mean	2427	7510	5123 (4852, 5395)	<0.001	2500	6725	4280 (3955, 4605)	<0.001

**Fig. 1:**
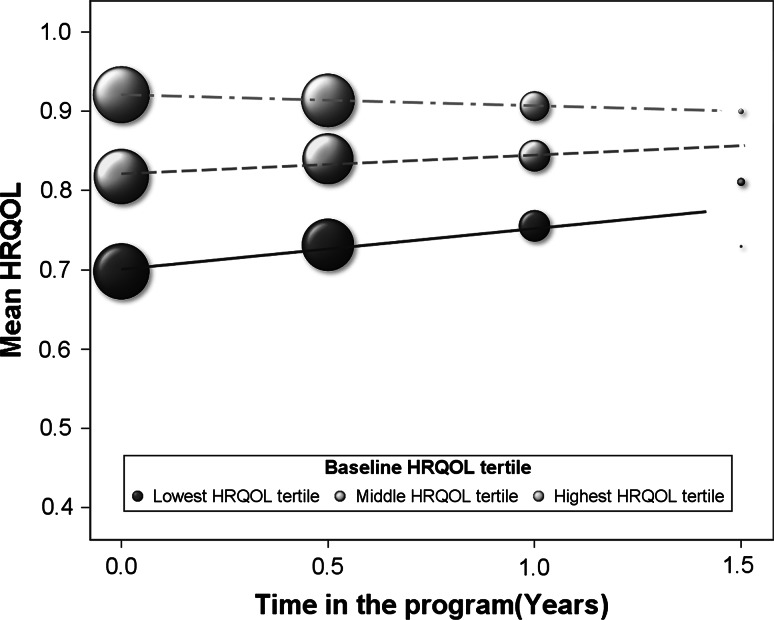
Temporal changes in HRQOL by baseline HRQOL tertile. *Note*: *Bubbles* represent the mean HRQOL level at baseline, at 6 month, and at 1 year of follow-up. The size of the bubbles is proportional to the number of assessments at each reporting time

Figure 1 and Table 3 show that temporal increases in HRQOL scores during follow-up were more pronounced among participants in the lowest tertile of baseline HRQOL score compared to those in the higher tertiles. Table 3 further shows that gender, body weight status, and changes in serum 25(OH)D were important and statistically significant determinants of improvement in HRQOL at 1 year of follow-up. The adjusted difference in mean temporal change was estimated to be 0.060 units higher among participants in the lowest relative to the highest tertile (Table 3). Those participants who realized increases in serum 25(OH)D of more than 50 nmol 25(OH)D/L had mean-adjusted temporal change that were 0.024 units higher as compared to those whose serum concentrations did not increase (Table 3). As per 25 nmol/L increase in 25(OH)D, HRQOL increased by 0.005 units, after adjusting for baseline HRQOL, baseline serum 25(OH)D, age, and gender (Table 3). Further, male participants experienced higher improvements in HRQOL compared to female participants and participants with obesity experienced lower improvements in HRQOL compared to normal weight participants. At 6 month of follow-up, changes in HRQOL were not statistically significantly different for weight status and increases in 25(OH)D but were different for baseline HRQOL tertile and gender (Table 3).

**Table 3:** Determinants of temporal changes in HRQOL of program participants at 6 months (*N* = 1795) and 1 year (*N* = 761)

Variable	Temporal change from baseline to 6 months (*N* = 1795)					Temporal change from baseline to 1 year (*N* = 761)				
	Mean change	Unadjusted		Adjusted^a^		Mean change	Unadjusted		Adjusted^a^	
		*β*	*p* value	*β*	*p* value		*β*	*p* value	*β*	*p* value
Gender										
Female (Ref.)	0.016	–	–	–	–	0.021	–	–	–	–
Male	0.019	0.003	0.531	0.010	0.030	0.032	0.011	0.142	0.016	0.020
Baseline age										
50–59	0.024	–	–	–	–	0.030	–	–	–	–
60–69	0.012	−0.012	0.060	−0.007	0.264	0.025	−0.005	0.583	0.005	0.518
70–79	0.021	−0.003	0.716	0.001	0.906	0.020	−0.010	0.358	−0.001	0.912
80+	0.012	−0.012	0.264	−0.014	0.160	0.025	−0.005	0.799	−0.017	0.320
Baseline income										
Other (Ref.)	0.013	–	–	–	–	0.021	–	–	–	–
Low income	0.020	0.007	0.148	−0.006	0.191	0.031	0.010	0.155	−0.005	0.478
Baseline serum 25(OH)D (100 nmol/L)	–	0.001	0.937	0.007	0.307	–	−0.018	0.056	−0.004	0.635
Baseline serum 25(OH)D (nmol/L)										
<25 nmol/L	0.078	0.066	0.054	0.041	0.197	0.093	0.055	0.273	0.042	0.361
≥25, <50 nmol/L	0.023	0.011	0.303	0.001	0.919	0.036	−0.002	0.874	−0.011	0.436
≥50, < 75 nmol/L (Ref.)	0.012	–	–	–	–	0.038	–	–	–	–
≥75, <100 nmol/L	0.021	0.009	0.185	0.008	0.189	0.022	−0.016	0.115	−0.006	0.523
≥100, <125 nmol/L	0.008	−0.004	0.590	−0.001	0.839	0.021	−0.017	0.106	−0.001	0.931
≥125 nmol/L	0.023	0.011	0.182	0.012	0.125	0.010	−0.028	0.017	−0.018	0.098
Baseline HRQOL score	–	−0.303	<0.001	−0.307	<0.001		−0.354	<0.001	−0.362	<0.001
Baseline HRQOL tertile										
Lowest HRQOL tertile (Ref.)	0.036	–	–	–	–	0.054	–	–	–	–
Middle HRQOL tertile	0.024	−0.013	0.033	−0.013	0.023	0.024	−0.030	<0.001	−0.031	<0.001
Highest HRQOL tertile	−0.008	−0.045	<0.001	−0.045	<0.001	−0.005	−0.059	<0.001	−0.060	<0.001
Baseline self-reported health										
Very good or excellent (Ref.)	0.014	–	–	–	–	0.023	–	–	–	–
Good	0.020	0.006	0.257	−0.018	<0.001	0.023	0.001	0.949	−0.025	0.001
Poor or fair	0.018	0.005	0.487	−0.049	<0.001	0.034	0.011	0.291	−0.047	<0.001
Baseline weight status										
Underweight or normal weight (Ref.)	0.011	–	–	–	–	0.024	–	–	–	–
Overweight	0.019	0.008	0.156	0.002	0.702	0.027	0.003	0.755	−0.003	0.666
Obesity	0.018	0.007	0.274	−0.007	0.236	0.020	−0.004	0.661	−0.024	0.008
Change in serum 25(OH)D (per 25 nmol/L)	–	−0.001	0.604	0.001	0.509	–	0.005	0.031	0.005	0.020
Change in 25(OH)D										
≤0 (Ref.)	0.027	–	–	–	–	0.017	–	–	–	–
>0, ≤25	0.017	−0.010	0.240	−0.001	0.862	0.021	0.004	0.740	0.013	0.243
>25, ≤50	0.014	−0.013	0.105	−0.004	0.611	0.025	0.008	0.474	0.017	0.138
>50	0.015	−0.012	0.143	−0.002	0.837	0.033	0.015	0.160	0.024	0.036

*Ref*. reference category, *25(OH)D* serum 25-hydroxyvitamin D concentrations

^a^Adjusted for age, gender, baseline HRQOL, and baseline 25(OH)D

When considering all determinants simultaneously, the HRQOL score increased by 0.023 (95 % CI 0.018, 0.028) per year (Table 4). When further considering vitamin D supplementation doses, the estimates in Table 4 did not substantially change. In addition, vitamin D supplementation dose had a minimal and not statistical significant effect on HRQOL because of collinearity of vitamin D supplementation doses and 25(OH)D concentrations and because vitamin D status is on the causal pathway. The observed increase of 0.023 in HRQOL per year translates into 0.012 QALYs gained per person per year. When change in 25(OH)D was added to the model, the effect of time in the program reduced slightly from 0.023 to 0.020 units per year and each 25 nmol/L increase in serum 25(OH)D was associated with a 0.002 increase in HRQOL (Table 4). This translates into 0.001 QALYs gained per 25 nmol/L increase in serum 25(OH)D per person per year.

**Table 4:** Determinants of temporal changes in HRQOL among 2119 program participants

Variable	Not including serum 25(OH)D		With serum 25(OH)D included	
	*β* (95 % CI)	*p* value	*β* (95 % CI)	*p* value
Years in program	0.023 (0.018, 0.028)	<0.001	0.020 (0.014, 0.026)	<0.001
Change in 25(OH)D (25 nmol/L)			0.002 (0.000, 0.004)	0.022
Baseline HRQOL	−0.178 (−0.197, −0.159)	<0.001	−0.179 (−0.198, −0.159)	<0.001
Gender				
Female (Ref.)	–	–	–	
Male	0.007 (0.003, 0.012)	0.002	0.007 (0.003, 0.012)	0.003
Baseline weight status				
Underweight or normal weight (Ref.)	–	–	–	–
Overweight	−0.001 (−0.006, 0.004)	0.716	−0.001 (−0.006, 0.004)	0.750
Obesity	−0.008 (−0.014, −0.002)	0.008	−0.008 (−0.014, −0.002)	0.009
Baseline age				
50–59 (Ref.)	–	–	–	–
60–69	−0.002 (−0.008, 0.004)	0.566	−0.002 (−0.008, 0.004)	0.538
70–79	−0.001 (−0.008, 0.006)	0.767	−0.001 (−0.008, 0.005)	0.730
80+	−0.008 (−0.018, 0.002)	0.138	−0.008 (−0.018, 0.002)	0.123

*Ref.* reference category, *25(OH)D* serum 25-hydroxyvitamin D concentration

## Discussion

This study revealed benefits of a real-world preventive health program that encourages vitamin D supplementation. Problems with mobility, usual activities, pain or discomfort, and depression or anxiety were all reported less at follow-up as compared to baseline. Overall HRQOL and QALYs gained during follow-up and paralleled improvements in vitamin D status.

The present study showed improvements in mobility and usual activities. This seems consistent with findings from observational studies and clinical trials that had reported benefits to mobility and functional status in terms of prevention of falls [12] and improvement in physical function [33]. A recent meta-analysis of randomized controlled trials concluded that vitamin D supplementation among unselected community-dwelling individuals may reduce the risk of skeletal or non-skeletal outcomes to up to 15 % [34]. In the present real-world evaluation, we observed reductions of 5.2 and 8.3 % in the reporting of problems with mobility and with usual activities, respectively. The very low prevalence of problems with self-care among program participants may be causing a ceiling effect whereby further improvements are difficult to achieve. Vitamin D has also been shown to be associated with less depression [17, 35] which seems consistent with our observation of a reduction in the reporting of problems with depression and anxiety. Of all dimensions, the largest reduction was reported for problems with “pain and discomfort” that dropped from 81.7 to 69 % in the first year. Bias by indication [36], whereby individuals with depression and anxiety and with pain and discomfort are more likely to participate and comply with program recommendations, may have contributed to a selective enrollment. At enrollment, we observed higher proportions of program participants reported problems with depression and anxiety (48 vs 32 %) and pain and discomfort (77 vs 70 %) compared to the general population of Alberta in the same age range [37].

As the present study is an evaluation of a real-world program rather than a randomized controlled trial with a strict protocol and blinded administration of the vitamin, it is not possible to disentangle the benefits of vitamin D supplementation from benefits of other program components such as promotion of healthy eating, active living, multivitamin use, and sun exposure. Therefore, and in light of the health promotion focus of this program evaluation, we had studied the importance of improving vitamin D status as a distinct achievement of the program. The regarding analyses revealed independent benefits to HRQOL arising from improvements in vitamin D status and from other program components.

The program effect translated into 0.012 QALYs gained per person per year, and the vitamin D status effect into 0.001 QALYs gained per 25 nmol/L increase in serum 25(OH)D per person per year. Assuming costs of CA$30 for an annual supply of vitamin D supplements, the cost-effectiveness ratio for someone who increases their vitamin D status with 50 nmol/L would be CA$15,000 per QALY ($30 divided by 2 times 0.001 QALY). The costs of the program in its pilot stage were estimated to range from CA $900 to 1200 per participant per year and for a sustained delivery of the scaled-up program CA $500 per participant per year. The cost-effectiveness ratio for the program is estimated to be CA$41,667 per QALY ($500/0.012 QALY). Both these ratios are considered cost-effective according to Rocchi et al. [38] who considered ratios below $50,000 per QALY as cost-effectiveness. In Canadian dollars in 2008, this is CA$114,000 per QALY. Canada and its provinces do not have an established cutoff threshold levels for cost per QALY and have a practice of considering other factors as well. A review of Canadian Common Drug Review concluded that new drugs were frequently accepted up to CA$80,000 per QALY though also noted that this threshold was not consistently applied [38]. Vitamin D supplementation is paid for by the individual, and the PN program is charitable. But even if vitamin D and the PN program were publicly funded, both ratios (program and vitamin D) would be cost-effective as the cost per QALY is in a range where most drugs are accepted. Moreover, it is important to note that HRQOL declines with age and the magnitude of this decline increases with age [39, 40]. Hazall et al. had estimated this natural (aging) decline in HRQOL to approximate 0.005 which translate in a reduction of 0.0025 QALY’s per person per year [39]. If we had contrasted the observed increases among program participants against the natural decline in HRQOL and QALY’s resulting from aging, the program and vitamin D benefits would have appeared even more favorable and more cost-effective.

The average serum 25(OH)D concentration of program participants was 93.4 nmol/L which is substantially higher than the average among Canadians aged 6–79 (67.7 nmol/L) and among Canadians 60–79 years of age (72.0 nmol/L) [9]. Self-selection of health-aware individuals interested to participate in the preventive health program may have contributed to this. As vitamin D status is positively related to HRQOL [20], and the present study revealed substantial larger program benefits for participants in the lower HRQOL tertile, one may expect larger benefits to similar interventions in populations that are better representative of Canadians. Where Health Canada recommendations apply to the general Canadian population, no earlier study reported on the benefits of improvements in vitamin D status for the quality of life in a general population. Though we conducted this in a community sample, caution is warranted when it comes from generalizing our findings as this sample may be self-selected toward a health-aware subpopulation and with a relatively high prevalence of problems with pain and discomfort [20]. Despite the large sample size, a second study limitation relates to the small number of participants that reported problems with self-care, which hampered some of the analyses. Further limitations relate to the limited duration of follow-up and the study design. The present study evaluated a real-world program. These results may therefore be particularly relevant for public health decision makers, but we acknowledged that stronger biological evidence comes from randomized controlled trials. We therefore recommend randomized controlled trials include pre- and post-intervention assessments of HRQOL to strengthen the biological evidence. A final limitation relates to the use of self-reported information which is prone to error, though the EQ-5D is an established and validated instrument [24] whereby the reporting error is expected to be limited given that it is short and easy to complete [26].

## References

[1] Holick MF (2007). Vitamin D deficiency. New England Journal of Medicine.

[2] Institute of Medicine, Food and Nutrition Board (2011). Dietary reference intakes for calcium and vitamin D.

[3] Health Canada. (2012). Vitamin D and calcium: Updated dietary reference intakes. Retrieved Oct 20, 2013, from http://www.hc-sc.gc.ca/fn-an/nutrition/vitamin/vita-d-eng.php

[4] Ekwaru JP, Zwicker JD, Holick MF, Giovannucci E, Veugelers PJ (2014). The importance of body weight for the dose response relationship of oral vitamin d supplementation and serum 25-hydroxyvitamin d in healthy volunteers. PLoS ONE.

[5] Zittermann A, Ernst JB, Gummert JF, Borgermann J (2014). Vitamin D supplementation, body weight and human serum 25-hydroxyvitamin D response: A systematic review. European Journal of Nutrition.

[6] van Groningen L, Opdenoordt S, van Sorge A, Telting D, Giesen A, de Boer H (2010). Cholecalciferol loading dose guideline for vitamin D-deficient adults. European Journal of Endocrinology.

[7] Vatanparast H, Calvo MS, Green TJ, Whiting SJ (2010). Despite mandatory fortification of staple foods, vitamin D intakes of Canadian children and adults are inadequate. Journal of Steroid Biochemistry and Molecular Biology.

[8] Prentice A (2008). Vitamin D deficiency: A global perspective. Nutrition Reviews.

[9] Langlois K, Greene-Finestone L, Little J, Hidiroglou N, Whiting S (2010). Vitamin D status of Canadians as measured in the 2007 to 2009 Canadian Health Measures Survey. Health Reports.

[10] Sohl E, van Schoor NM, de Jongh RT, Visser M, Deeg DJ, Lips P (2013). Vitamin D status is associated with functional limitations and functional decline in older individuals. Journal of Clinical Endocrinology and Metabolism.

[11] Murad MH, Elamin KB, Abu Elnour NO, Elamin MB, Alkatib AA, Fatourechi MM, Almandoz JP, Mullan RJ, Lane MA, Liu H, Erwin PJ, Hensrud DD, Montori VM (2011). Clinical review: The effect of vitamin D on falls: A systematic review and meta-analysis. Journal of Clinical Endocrinology and Metabolism.

[12] Bischoff-Ferrari HA, Dawson-Hughes B, Staehelin HB, Orav JE, Stuck AE, Theiler R, Wong JB, Egli A, Kiel DP, Henschkowski J (2009). Fall prevention with supplemental and active forms of vitamin D: A meta-analysis of randomised controlled trials. BMJ.

[13] Janssen HC, Samson MM, Verhaar HJ (2002). Vitamin D deficiency, muscle function, and falls in elderly people. American Journal of Clinical Nutrition.

[14] Bischoff-Ferrari HA, Willett WC, Orav EJ, Lips P, Meunier PJ, Lyons RA, Flicker L, Wark J, Jackson RD, Cauley JA, Meyer HE, Pfeifer M, Sanders KM, Stahelin HB, Theiler R, Dawson-Hughes B (2012). A pooled analysis of vitamin D dose requirements for fracture prevention. New England Journal of Medicine.

[15] Sahota O (2010). Reducing the risk of fractures with calcium and vitamin D. BMJ.

[16] Lips P, Wiersinga A, van Ginkel FC, Jongen MJ, Netelenbos JC, Hackeng WH, Delmas PD, van der Vijgh WJ (1988). The effect of vitamin D supplementation on vitamin D status and parathyroid function in elderly subjects. Journal of Clinical Endocrinology and Metabolism.

[17] Anglin RE, Samaan Z, Walter SD, McDonald SD (2013). Vitamin D deficiency and depression in adults: Systematic review and meta-analysis. British Journal of Psychiatry.

[18] Ju SY, Lee YJ, Jeong SN (2013). Serum 25-hydroxyvitamin D levels and the risk of depression: a systematic review and meta-analysis. J Nutr Health Aging.

[19] Huang W, Shah S, Long Q, Crankshaw AK, Tangpricha V (2013). Improvement of pain, sleep, and quality of life in chronic pain patients with vitamin D supplementation. Clinical Journal of Pain.

[20] Chao YS, Ekwaru JP, Ohinmaa A, Griener G, Veugelers PJ (2014). Vitamin D and health-related quality of life in a community sample of older Canadians. Quality of Life Research.

[21] Chao YS, Brunel L, Faris P, Veugelers PJ (2013). The importance of dose, frequency and duration of vitamin D supplementation for plasma 25-hydroxyvitamin D. Nutrients.

[22] Chao YS, Brunel L, Faris P, Veugelers PJ (2013). Vitamin D status of Canadians employed in northern latitudes. Occup Med (Lond).

[23] Heaney RP, French CB, Nguyen S, Ferreira M, Baggerly LL, Brunel L, Veugelers P (2013). A novel approach localizes the association of vitamin D status with insulin resistance to one region of the 25-hydroxyvitamin D continuum. Adv Nutr.

[24] Oemar M., & Janssen B. (2013). EQ-5D-5L user guide basic information on how to use the EQ-5D-5L instrument.

[25] Herdman M, Gudex C, Lloyd A, Janssen M, Kind P, Parkin D, Bonsel G, Badia X (2011). Development and preliminary testing of the new five-level version of EQ-5D (EQ-5D-5L). Quality of Life Research.

[26] Tidermark J, Zethraeus N, Svensson O, Tornkvist H, Ponzer S (2002). Femoral neck fractures in the elderly: functional outcome and quality of life according to EuroQol. Quality of Life Research.

[27] Harris, M. (2013). Digital divide persists in Canada, both in access and Internet fluency. Financial Post.

[28] World Health Organization. (2013). Obesity and overweight. Retrieved Oct 2013, from http://www.who.int/mediacentre/factsheets/fs311/en/index.html

[29] Holick MF, Binkley NC, Bischoff-Ferrari HA, Gordon CM, Hanley DA, Heaney RP, Murad MH, Weaver CM, Endocrine S (2011). Evaluation, treatment, and prevention of vitamin D deficiency: An Endocrine Society clinical practice guideline. Journal of Clinical Endocrinology and Metabolism.

[30] Vieth R, Bischoff-Ferrari H, Boucher BJ, Dawson-Hughes B, Garland CF, Heaney RP, Holick MF, Hollis BW, Lamberg-Allardt C, McGrath JJ, Norman AW, Scragg R, Whiting SJ, Willett WC, Zittermann A (2007). The urgent need to recommend an intake of vitamin D that is effective. American Journal of Clinical Nutrition.

[31] Health Quality Council of Alberta. (2013). Alberta Provincial Norms for EQ-5D-3L. http://www.hqca.ca/assets/files/Alberta%20Provincial%20Norms%20for%20EQ-5D%203L(1).pdf

[32] Whitehead SJ, Ali S (2010). Health outcomes in economic evaluation: the QALY and utilities. British Medical Bulletin.

[33] Bunout D, Barrera G, Leiva L, Gattas V, de la Maza MP, Avendano M, Hirsch S (2006). Effects of vitamin D supplementation and exercise training on physical performance in Chilean vitamin D deficient elderly subjects. Experimental Gerontology.

[34] Bolland MJ, Grey A, Gamble GD, Reid IR (2014). The effect of vitamin D supplementation on skeletal, vascular, or cancer outcomes: A trial sequential meta-analysis. Lancet Diabetes Endocrinol.

[35] Polak MA, Houghton LA, Reeder AI, Harper MJ, Conner TS (2014). Serum 25-hydroxyvitamin D concentrations and depressive symptoms among young adult men and women. Nutrients.

[36] Signorello LB, McLaughlin JK, Lipworth L, Friis S, Sorensen HT, Blot WJ (2002). Confounding by indication in epidemiologic studies of commonly used analgesics. American Journal of Therapeutics.

[37] Health Quality Council of Alberta. (2014). Alberta population norms for EQ-5D-5L. http://hqca.ca/studies-and-reviews/health-outcomes-measurement/2014-alberta-population-norms-for-eq-5d-5l/

[38] Rocchi A, Menon D, Verma S, Miller E (2008). The role of economic evidence in Canadian oncology reimbursement decision-making: to lambda and beyond. Value Health.

[39] Hazell ML, Morris JA, Linehan MF, Frank TL (2009). Temporal change in health-related quality of life: A longitudinal study in general practice 1999–2004. British Journal of General Practice.

[40] Burstrom K, Johannesson M, Rehnberg C (2007). Deteriorating health status in Stockholm 1998–2002: Results from repeated population surveys using the EQ-5D. Quality of Life Research.

